# Varicose Veins

**Published:** 1829-05

**Authors:** 


### VARICOSE VEINS.

A CORRESPONDENT suggests that the best mode of intercepting the circulation through the veins, in cases of ulcerated legs, for example, would be by applying a ligature to the vein in the usual manner, and then dividing the vein above the ligature. By this proceeding, the superior portion of the vein, at the place where it is intercepted, would be no more likely to communicate an irritation to the heart than in cases of amputation, when the same vein would of course be divided. The ligature remaining on the lower portion of the vein would, it is presumed, be sufficient to prevent any considerable hemorrhage; since no blood could escape from the superior orifice except by a retrograde circulation, which the valves of the vein would so far oppose, that a common dressing, with the aid of a compress, would be a sufficient security. It is agreeable with analogy to suppose that the ligature may be suffered to remain on the lower orifice of the vein until it is spontaneously detached, without danger of any sympathetic affection of the heart.

